# Colliding micro-shock waves

**DOI:** 10.1038/s41598-026-54605-x

**Published:** 2026-06-11

**Authors:** Lars Jepsen, Walter Garen, Ulrich Teubner

**Affiliations:** 1 Institute for Laser and Optics, University of Applied Sciences Emden-Leer, Constantiaplatz 4, 26723 Emden, Germany; 2 Institute of Physics, Carl von Ossietzky University Oldenburg, Carl-Von-Ossietzky-Straße 11, 26129 Oldenburg, Germany

**Keywords:** Shock waves, Colliding shocks, Shock probing, Shock tube, Shock reflection, Laser induced shocks, Hot flow, Boundary layer, Engineering, Optics and photonics, Physics

## Abstract

Shock waves, disturbances that propagate with supersonic velocity in a fluid, are prevalent in nature and across nearly all natural sciences. They find diverse applications in fields such as medicine, aerospace engineering and physical chemistry, where experiments are conducted mostly in macroscopic tubes with an inner diameter ranging from more than 1 mm up to the meter scale. While the theoretical framework for macroscopic shock waves is well-established, the behavior of shock waves in capillaries with diameters in the micrometer range—referred to as “micro-shock waves”—remains largely unexplored. This paper presents novel experimental investigations on the collision of shock waves in micro-capillaries, a fundamental research that has never been done before. These investigations, involving both steady and unsteady drivers, are of significant importance for shock wave physics in general, especially given the limited research on unsteady shock wave collisions. Even more, they play a crucial role in the analysis of micro-shock waves, since they contribute to a more complete characterization of the post-shock region. With the growing interest in microfluidic devices, this research is also important to advance the understanding of supersonic flows at the microscale. Even in the application of high-repetition-rate laser sources, micro-shock wave physics is involved.

## Introduction

The subject of shock waves is one of the most interdisciplinary fields. Among the wide variety of shock waves, astrophysical shocks^1,2^ are by far the largest. On the other hand, shock waves, and even their collision, can be studied experimentally on a very small scale, for example using laser-produced plasmas (LPP). Typically, a pulsed laser is strongly focused to a small spot, producing a hot and relatively dense plasma^3^ and potentially emitting a shock wave. Due to the short duration of the laser pulse, the laser-plasma as driver of the shock wave is considered unsteady and the shock is, in principle, similar to a blast wave (note that, despite frequent use of this term, we consider blast waves as a particular type of shock wave). Numerous studies have addressed this topic (see, e.g.^ 4^ and as a recent example^ 5^). However, both the aforementioned astrophysical shocks and the laser-induced shocks propagate and expand freely, without any artificial confinement. Particularly, in the case of LPP, the shock wave expands spherically and is commonly referred to as a Sedov-Taylor blast wave.

Fundamentally differing from this scenario is the propagation of planar shock waves in shock tubes. These tubes are basic tools for experiments on shock wave phenomena in gas dynamics and restrict the propagation geometrically. In many cases, generation with a steady driver and confinement within a tube lead to the propagation of well-directed shock waves with significantly enhanced propagation distances compared to the unconfined geometry. Thus, the extreme conditions of pressure *p*, temperature *T* and density $\rho$, which occur rapidly behind the shock front, can be utilized for experiments such as those mentioned above in aerospace or kinetic processes in physical chemistry, including combustion. For all those experiments, typically the inner tube diameter *D* is macroscopic, i.e., ranging from 1 mm up to more than a meter.

When focusing on shock waves propagating in tubes - this work will restrict to this - nearly all research is related completely to the macroscopic regime, corresponding to the previously mentioned range of *D*. These shock waves will be termed “macro-shock waves”, even though phenomena on the micro-scale may still play a role. Understanding the micro-world will enable new potential, in terms of both, fundamental research and applications such as microfluidic technological, biological, and medical devices or even atomic physics where high-repetition-rate Lasers create pressure and shock waves in confined geometries.

Consequently, shock waves that propagate in the confined geometry of a small capillary, with *D* in the order of tens of micrometers and, for instance, up to 300 $\upmu$m, represent a novel branch of fluid physics, the field of “micro-shock waves”. The novelty arises from the fact that there is no generally applicable trivial down-scaling and, e.g., shock wave research can be extended to regimes that are inaccessible with macroscopic shock tubes.

Theoretical investigations on micro-shock waves have been performed by several groups^6–13^. We highlight the work of Brouillette^6^, who introduced a dimensionless scaling parameter $S_c$ that has been relevant to many subsequent studies on micro-shock waves. This parameter links physical quantities important for the micro-scale regime: The tube diameter *D*, the Reynolds number $R_e$, and the hot flow length $l_h$ (details are not discussed here): $S_c = (R_e \cdot D)/(4 \cdot l_h)$. It is then used to scale a correction term in the RH relations, trying to account for the scale-dependent effects on the Mach number observed in micro-scale experiments. However, experimental work has been absent for a long time, due to the difficulty of generating shocks in tubes with very small diameters. Despite considerable interest, the first experiments were not conducted until 2009 to 2012, when Mirshekari and Brouillette presented first results obtained with a micro tube having a hydraulic diameter of 34 $\upmu$m^14,15^. Even later, experimental work was limited to a few studies only, as by Giordano et al.^16^, Janardhanraj et al.^17^ and those of the authors group^18–20^. Nevertheless, significant progress has been achieved, for example, the first experimental measurement of the hot flow length^19^.

A common finding across both, theoretical and experimental investigations is that classical shock wave theory, which accurately describes macro-shock waves^21,22^, fails to capture the behavior of micro-shock waves (with some exceptions). In contrast to macro-shock waves, for which details of shock wave propagation and attenuation effects usually are neglected, this is not possible in the case of micro-shock waves. In the latter, the normalized maximum propagation length *L*/*D* (with *L* being the tube length) can exceed that for macro-shock waves by more than an order of magnitude. Moreover, the shocks were found not to be restricted to the turbulent regime, but a change to the laminar regime was observed and, of much importance, the boundary layer developing behind the shock front can reach a significant thickness with respect to *D*. The first estimate for the boundary layer thickness $\delta \approx \sqrt{\nu \cdot t_{h}}$ was introduced by^23^, where $\nu$ denotes the viscosity and $t_h$ the time scale associated with $l_h$. There have been later works on boundary layer effects, e.g., in case of mm-shock waves and also empirical adjustments to boundary layer growth were made. References^24–27^ may serve as examples. Nevertheless, further and particularly more detailed measurements have not yet been performed. Consequently, conducting suitable experimental work remains a challenge. There is a need to catch up with ongoing theoretical work as in ^28^ and to push micro-shock wave research with experiments that are completely different from any previous ones.

From this perspective, research on micro-shock waves may also provide deeper insight into general shock wave physics, particularly with shock tubes of any value of *D* and *L*, and for both steady and unsteady drivers. Investigations of the head-on collision of two micro-shock waves of equal strength presents such an opportunity. In contrast to studies of shock wave reflection at the closed end of a tube, reflection from the symmetric collision is, at best, lossless. This is the situation of an ideal reflector which allows to learn more about the shock wave interaction itself. This is potentially also useful for applications such as the study of non-equilibrium rates of relaxing gases^29^. Although numerous studies on shock wave collision have been conducted in the macro scale, only some of them employ shock tubes, and none involve small-diameter tubes (see, e.g., the review articles^2,30^; note that oblique shocks, which are of considerable interest for applications in aerospace technology, are entirely disregarded in the present work). Exceptions are the experiments with a “double shock wave tube” (with *D*$\approx$ cm) of Garen and Lensch^29^ and the development of a counter-driven shock tube of similar diameter by Tamba et al.^31^. However, related collision experiments have not been conducted so far.

In the present experimental work, we report major achievements on shock wave research, specifically in the field of micro-shock waves:

*First*, we report on the collision of shock waves in micro-capillaries ($D = 100~\upmu \textrm{m}$ and $200~\upmu \textrm{m}$), marking the first experiments ever conducted in this context.

The *second* significant progress is the comparison of colliding shocks generated by a steady and an unsteady driver, respectively, under otherwise identical experimental conditions. To the best of our knowledge, such a study has never been performed before. This is unique even for macro-shock waves. In this way, the present work also contributes substantially to the field of collision of blast waves, as the collision of unsteady shock waves remains remarkably limited (see statement in a recent review article^30^). In addition, the present experiments provide potential to investigate collisions of shocks of different strengths (“asymmetric shocks”) or with different driver steadiness (one steady and the other unsteady).

The *third* important novelty is the experimental access to the temperature directly behind a micro-shock wave front. Such direct or indirect measurements are difficult for any shock wave and have never been done for micro-shocks. This is achieved by using the shock reflected from the collision as a probe for the incident shock region. Changing the collision position, in principle, this approach enables a characterization of the shock region at any axial position *x* and at any time *t*. This is of particular importance for the further investigation of the boundary layer regions within micro-shock wave experiments such as the presented ones. However, we note that a comprehensive investigation of micro-shock wave physics, including modeling or scaling with respect to *D*, *M*, or other parameters, is well beyond the scope of the present work.

## Results

### Scheme and definitions

**Fig. 1:**
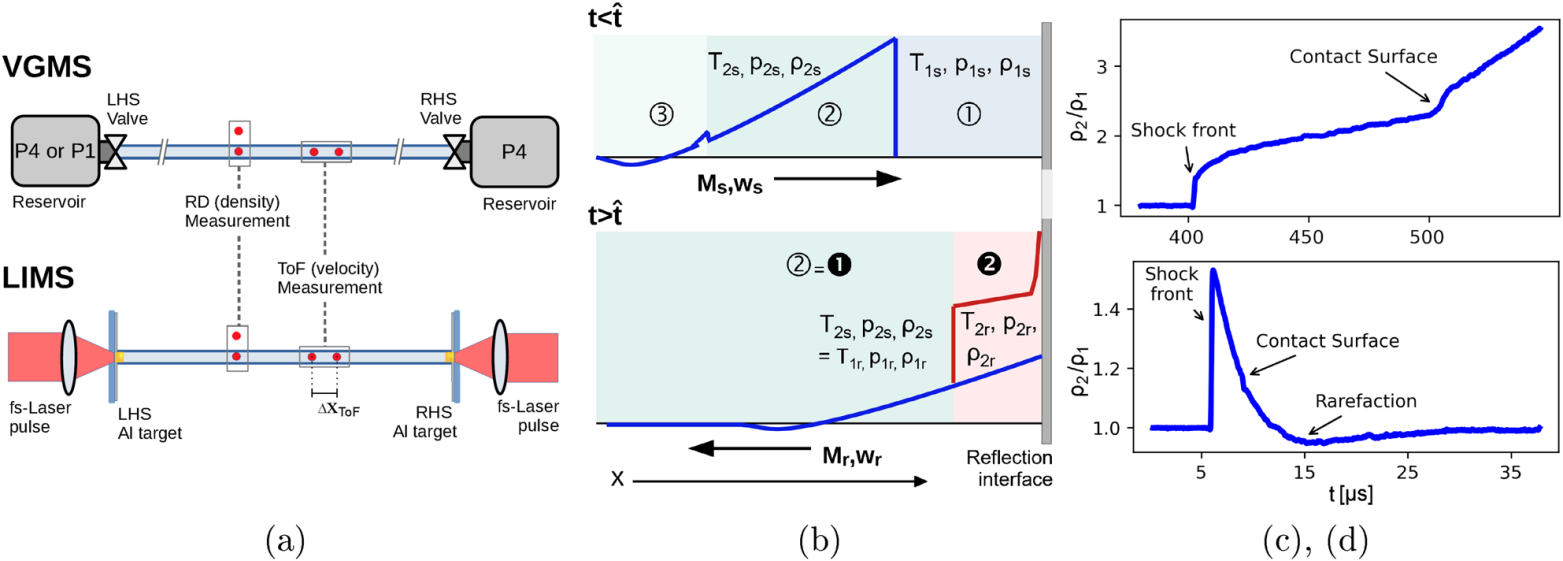
(**a**) Scheme for the measurements of the micro-shock wave collisions. The driver on the LHS and the RHS, respectively, can be chosen independently. This could be either an unsteady driver (LIMS) or steady driver (VGMS). The size of the reservoir is not to scale. The optical diagnostic is illustrated by the two laser interferometer beams (perpendicular to the paper) for the two operation modes. (**b**) Definition of the states and regions for the incident and the reflected shock. Note that regions $\textcircled {2}$ and ❶ are the same, while regions $\textcircled {1}$ and ❷ differ significantly. (**c**) Measured density as a function of time for LIMS (**c**) and VGMS (**d**) (RD-measurements).

The scheme of the head-on collision of micro-shock waves is illustrated in Fig. 1a. The shock waves are initiated at both ends of a high-quality glass capillary with a square cross-section of diameter $D = 100~\upmu \textrm{m}$ and $200~\upmu \textrm{m}$ (in the following we will not differentiate between widths and diameters; here geometric and hydraulic diameter are the same). This was achieved either by means of a particular femtosecond-LPP (fs-LPP) with an adapted geometry^18^ or by means of a fast magnetic valve^19,20^. In the following we will distinguish between “laser-plasma-induced micro-shock waves” (LIMS) and “valve-generated micro-shock waves” (VGMS).

For LIMS the test gas was air at laboratory environmental conditions of $T = 296~\textrm{K}$, $p = 1015~\textrm{hPa}$, while different gases and pressures were used for VGMS. Although, in principle, the driver strength on both sides could be set differently, for the present experiments it was set equally to obtain a symmetric collision in the middle of the capillary, i.e., at $x = L/2$. This could be arranged perfectly in the case of LIMS, as both sides are driven by the same laser pulse. Therefore, timing and strength are the same on both sides and any relative fluctuation is avoided. While the valve characteristics for VGMS from both sides could differ slightly, it was verified within the experiment that the trajectory *M*(*x*) for the shock wave from the left-hand side (LHS) and from the right-hand side (RHS) is the same, apart from the direction. The timing jitter between both is below $1~\upmu \textrm{s}$, which is keeping the *x*-position where both shocks collide within a range of $\hat{x} = L/2 \pm 0.3~\textrm{mm}$. For the discussion we would also like to define the position where the shock front arrives at a given time *t* as $x_s(t)$ and the time when the shock front arrives at a given position *x* as $t_s(x)$.

The definition and indices of the parameters *p*, $\rho$, *T*, etc., comply with the standard conventions: Index 1 refers to the region $\textcircled {1}$ in front of the incident shock and index 2 to the region $\textcircled {2}$ behind the shock front (see Fig. 1b). Moreover, the index *s* is used for the incident shock wave and *r* for the reflected one. Since region $\textcircled {1}$ is undisturbed, $p_{1s}$, $\rho _{1s}$, $T_{1s}$, and $a_{1s}$ are constant and given by the laboratory environmental conditions. When the particle velocity $u_p = 0$, $w_s$ is the absolute velocity of the shock wave. Consequently, with $a_{1s}$ as the sound velocity, one obtains the Mach number of the incident shock wave $M_s = w_s / a_{1s}$. If $u_p \ne 0$, the shock wave velocity v is given in the laboratory frame.

Parameters directly related to the collision and reflection are marked with ‘^’; therefore, the time when the collision occurs is $\hat{t}$ and the related spatial position is $\hat{x}$ (equal to *L*/2). For physical correctness, it should be noted that whenever the collision point is addressed, the conditions for $M_r$ and $u_p$ refer to an infinitesimal time or distance after the reflection, since exactly at $\hat{x}$ the particle velocity must be zero and the reflection has not yet formed. Immediately after the collision, the region behind the incident shock (region $\textcircled {2}$) is identical to region ❶ in front of the reflected shock. For simplicity, all velocity-related values are given as magnitudes.

The micro-shock waves are analyzed by an advanced laser interferometer with two operation modes (Fig. 1a). Briefly (details in Methods), the first method directly measures the shock velocity *w* using a light barrier principle (time-of-flight measurement, ToF). The second method yields the local density $\rho _{2}$ inside the capillary relative to the reference density $\rho _{1}$ outside (RD-measurement). This allowed to deduce the density jump at the shock front $\rho _{2}/\rho _{1}$ directly. Examples for density traces are shown in Fig. 1c and Fig. 1d for shocks driven by a steady and an unsteady driver. Consequently, from a series of successive measurements at different *x*-positions, the spatial evolution of the shock wave velocity $w_s(x)$ and that of the density ratio $\rho _2/\rho _1(x)$ were determined.

In general, it has been observed that the Mach number *M* of a micro-shock wave is relatively moderate. Depending on the specific experiment, *M* typically ranges from 1 to 3 and is therefore smaller than in comparable macro-shock waves experiments with the same initial conditions^6,18–20,32^. An exception is the initial phase of LIMS, where *M* can reach values larger than 10^18^. However, in the present work, we deliberately restrict to weak shocks in order to avoid high-temperature effects and real-gas behavior. In this regime, the temperature increase across the shock remains moderate, and the specific heat ratio $\gamma$ can be assumed constant to a good approximation.

### Conditions and propagation

**Fig. 2:**
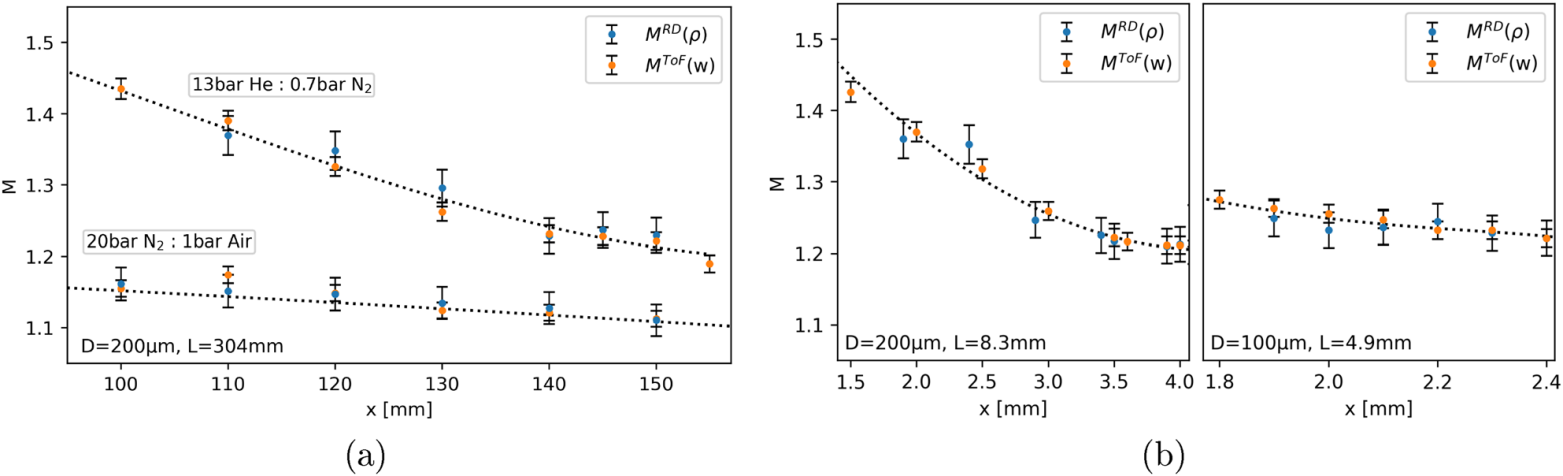
VGMS in a $D=200\,\upmu$m capillary and different gas compositions (**a**) and LIMS in $D=200\,\upmu$m and $D=100\,\upmu$m capillaries (**b**). Mach number evolution of a single-side generated micro-shock wave measured by the ToF-method and the RD-method, respectively. The Mach number at the collision position (at the very right) was set to $\hat{M} = 1.2$. Although $\hat{M}$ is the same for all displayed situations, the driver conditions and diameters in (**a,b**) lead to a different attenuation. Thus *L* and $\hat{x}=L/2$ differ strongly in absolute values (see Table 1). Dashed lines are to guide the eye.

Prior to the collision experiments, single shock wave generation and propagation was investigated. The present work restricts to the vicinity of the region where later the collision occurs (for the full range see^18,19^). Although not new, it is important to note that under the present experimental conditions, the Mach number obtained from the ToF-measurement, $$M_s^{ToF} = w_s / a_{1s}$$, and that derived from $$\rho _{2s}/\rho _{1s}$$ (RD-measurement) using the Rankine-Hugoniot relations (RH)^21,22^, $$M_s^{RD}$$, do not differ within the experimental error in the region of interest near $$\hat{x}$$ (Fig. 2). For the range $$x \le \hat{x}$$, this is crucial for the following collision experiments. This result was expected due to the low value of $$M_s$$, particularly if the flow in the micro-capillary is, to good approximation, regarded as one-dimensional (thread flow). For the present conditions, the Knudsen number in the core flow is well within the continuum regime (we may estimate $$K_n< 10^{-3})$$, supporting the validity of the Rankine–Hugoniot description. While a local change of the Knudsen number may occur in boundary layers of the capillary or further behind the shock front^7^, the influence on the (measured) shock jump is expected to be negligible. An estimate of the scaling parameter $$S_c$$ for the VGMS yields a value close to unity ($$S_c \approx 0.95$$), consistent with the observed agreement between the ToF-based and RH-based Mach numbers. It should be noted that the simplified RH model (1D, without friction or heat) may not necessarily apply for higher Mach numbers or smaller capillary diameters. Additional single shock wave generation measurements at higher Mach numbers ($$M >1.5$$) indicate increasing deviations from RH, consistent with the expected breakdown of the simplified model, though these data are not shown here and will be discussed in future work.

Although this work emphasizes the collision of micro-shock waves, it is still important to briefly describe the conditions for the incident shocks before the collision and to provide a comparison of the shock generation methods, LIMS and VGMS. In principle, a comparison of shock waves under different conditions, such as different gases, pressures or tube diameters, could be made by keeping the initial Mach number the same. However, for the present work, the Mach number is fixed at the collision position. Specifically, the Mach number was set to a value as close as possible to $\hat{M}_s = 1.20$ for nearly all of the experiments. This value is low enough to fall within a regime well-described by RH, which is essential for comparison with existing applicable theory. At the same time, it is still high enough to maintain the shock reflection in the supersonic regime.

For the VGMS-measurements, a $D = 200~\upmu \textrm{m}$ capillary with different driver/test gas combinations and different pressures was used. Fig. 2 shows an example of experimental results, including those from the data of 13 bar He into 0.7 bar $\text {N}_2$, for which the final decision was made. For the collision experiments, the capillary length *L* was chosen so that, after propagating a distance of $x = L/2$, the shock Mach number decayed to the intended value of $\hat{M}_s^{ToF} = 1.20$. For practical reasons, all VGMS-measurements were done with the same $L = 304~\textrm{mm}$ (both collision measurements and those with single-side shock generation). The related attenuation is strongly influenced by friction effects and also the interplay of the post-shock region with the contact surface. Moreover, boundary layer effects play a crucial role, particularly in the case of steady-driven micro-shock waves, where the boundary layer may occupy a significant portion of the capillary cross-section. Depending on the applied model and propagation length, this could be around 45-65% of the cross section in the experiment with the 200$\upmu$m capillary^20^. Particularly for VGMS, the obtained density further behind the shock front may be slightly increased due to boundary layer growth. Although the present results would permit an even more detailed analysis of these processes, such an investigation is beyond the scope of this work. Related studies are discussed elsewhere (see, e.g. ^6,7,9,10^) and will not be repeated here.

The complementary method to VGMS is LIMS. The two methods differ fundamentally in that VGMS provides a steady driver, whereas LIMS generates an unsteady driver. Furthermore, due to increased coupling losses at the interface between the valve and the capillary, experiments with very small diameters, below $D = 200~\upmu \textrm{m}$, are performed using LIMS (a more general discussion can be found in the review article^33^). In the present work, the LIMS experiments were conducted using capillaries with $D = 100~\upmu \textrm{m}$ and $D = 200~\upmu \textrm{m}$, respectively, and a fixed laser intensity of $I_L = 3\times 10^{12}~\mathrm {W/cm^2}$ for both diameters (Fig. 2b). During propagation, the initial shock strength decreases (mainly due to core flow expansion^18^). At the collision position $\hat{x} = L/2$, the Mach number $\hat{M}_s^{ToF} = 1.19$ was measured for the capillary with $D = 200~\upmu \textrm{m}$ and $L = 8.3~\textrm{mm}$, while for the capillary with $D = 100~\upmu \textrm{m}$ and $L = 4.9~\textrm{mm}$, $\hat{M}_s^{ToF} = 1.21$ was achieved. Experiments with shorter capillaries and consequently higher collision Mach numbers have been carried out in addition. The complete parameter set, with error estimation, is summarized in Table 1.

### Micro-shock collision and reflection: (a) LIMS

**Fig. 3:**
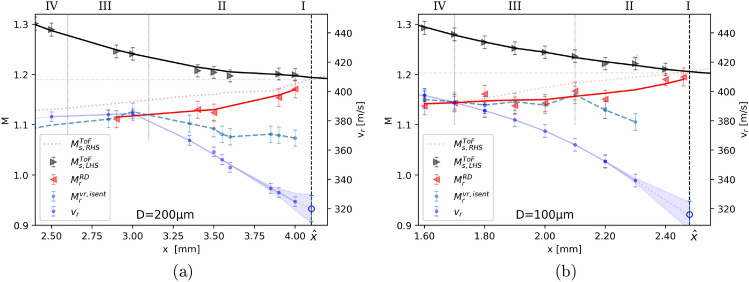
Results from colliding LIMS experiments in (**a**) $D = 200\,\upmu$m and (**b**) $D = 100\,\upmu$m capillaries. Due to symmetry, only the reflection of the incident shock from the LHS is shown (black solid symbols and line, $M^{ToF}_{s,LHS}$). The reflection at $x=\hat{x}$ yields $M_r^{RD}$ (red solid symbols and line). The velocity $\textrm{v}_r$ of the reflected shock and its extrapolation to $x=\hat{x}$ are related to the laboratory frame (blue solid line). Assuming isentropic conditions, $M_r^{\textrm{v}r,isent}$ (cyan line) is calculated from $\textrm{v}_r$ (see text). For comparison, the dotted red line shows the evolution of the single-side generated RHS shock when propagating into undisturbed conditions. All lines are intended to guide the eye. Regions indicated by Roman numerals are discussed in the text.

In principle, the collision experiments and the analysis are conducted in a same manner as previously. However, a short discussion is needed: The incident shock wave propagates into the undisturbed region $\textcircled {1}$ (laboratory environment), while the reflected shock travels into region ❶ which has already been influenced by the incident shock. Thus, $a_{1r} = a_{2s}$, $\rho _{1r} = \rho _{2s}$, $T_{1r} = T_{2s}$, etc., are now functions of *x* and *t*, respectively. Consequently, the sound velocity $a_{1r}$ is a function of $T_{2s}(x,t)$, whereas $a_{1s}$ is a given by the constant laboratory conditions. Furthermore, the density jump $\rho _{2r}/\rho _{1r}$ at the reflected shock (RD-measurement) occurs on a background given by $\rho _{2s}(x,t)$, which must be taken into account for any analysis. Additionally, the gas behind the incident shock is no longer at rest, which influences the observed $w_r$. Thus the ToF-measurement yields the velocity $\textrm{v}_r$ relative to the laboratory frame (see below).

Figure 3a shows the experimental data from a LIMS experiment with a symmetric shock collision inside a capillary of diameter $D = 200~\upmu \textrm{m}$ and length $L = 8.3~\textrm{mm}$. The data points of the incident shock Mach number are represented by black triangles pointing to the right (according to the propagation direction). The Mach number at the collision position was $\hat{M}_s^{ToF} = 1.19 \pm 0.01$. Unlike the shock reflection at the end wall of a tube, the collision of two identical shock waves can be assumed to be ideal to very good approximation. Hence, the preservation of momentum and the RH relations can be used to describe $\hat{M}_r$ as a function of $\hat{M}_s$. This is particularly due to the low value of $\hat{M}_s$. Therefore, for the present condition, the expected reflected Mach number can be calculated from Eq. (5) as $\hat{M}_r(\hat{M}_s = 1.19) = 1.18$ (see “Methods”).

For easier discussion, within region $\textcircled {2}$ we define region I as the immediate proximity of the shock front, which extends from $\hat{x} - \epsilon$ to $\hat{x}$, where ideally $\epsilon \rightarrow 0$. Regions II to IV, which are located in region $\textcircled {2}$ as well, are defined according to Figs. 3a, b (see “Discussion”).

The reflected shock propagates into region $\textcircled {2}$, thus into a region which has already been altered by the incident shock. Taking into account the related background, $M_r$ was estimated from the RD-measurement data together with the RH relations (Fig. 3a, red triangles pointing to the left). The obtained Mach number $\hat{M}_r^{RD} = 1.18$ is in good agreement with the theoretical value estimated above.

The velocity of the reflected shock is directly obtained from the ToF-measurement (Fig. 3a, data displayed in blue). It decreases rapidly for $x > 3~\textrm{mm}$, as $x \rightarrow \hat{x}$. This is a result of the modified conditions of *p*, $\rho$ and *T* in the post-shock region $\textcircled {2}$. The reflected shock propagates into region ❶ = $\textcircled {2}$, where the particle velocity $u_{ps}$ is affected by the related conditions. This is in contrast to region $\textcircled {1}$, where $u_{ps} = 0$. Therefore, to obtain the velocity $w_r$ of the reflected shock relative to the gas flow, the following correction must be made: $w_r = \textrm{v}_r + u_{ps}$

Due to the good agreement between the Mach numbers obtained from the ToF- and the RD-measurement and due to the low Mach numbers, namely $M_s^{ToF}=M_s^{RD}$ (Fig. 2), the RH relations are applied on $\hat{M}_s$, to calculate the particle velocity $u_{ps}$ and the temperature $\hat{T}$ at $\hat{x}$ (details in Methods). With $\hat{M}_s^{ToF}=1.19$, this yields $\hat{u}_{ps}^{RH}=101$ m/s and $\hat{T}_{2s}^{RH}=330$ K. Consecutively, the sonic speed directly behind the front of the incident shock, and rather close to $\hat{x}$, can be determined from its temperature dependence: $\hat{a}^{RH}=a_{2s}=a_{1r}=364$ m/s (Eq. 4). In turn the reflected shock speed based on RH results in $\hat{w}_{r}^{RH}=\hat{M}_r^{RD} \cdot \hat{a}_{1r}^{RH}=429$ m/s.

Although the finite distance $\Delta x_{\textrm{ToF}}$ (see Methods) prevents a direct measurement of $\textrm{v}_r$ very close to $\hat{x}$, the extrapolation to $\hat{\textrm{v}}_r=320$ m/s appears reliable, despite the uncertainty introduced by extrapolating data from the incident post-shock region. Consider the blue shaded area in Fig. 3a as a reasonable estimation within $\pm 5$%. With the previously calculated $\hat{u}_{ps}^{RH}$ this results in $\hat{w}_{r}^{vr,RH}=421$m/s which is in good agreement with $\hat{w}_{r}^{RH}$(429 m/s). In total, this gives again a proof of consistence within the data analysis.

**Fig. 4:**
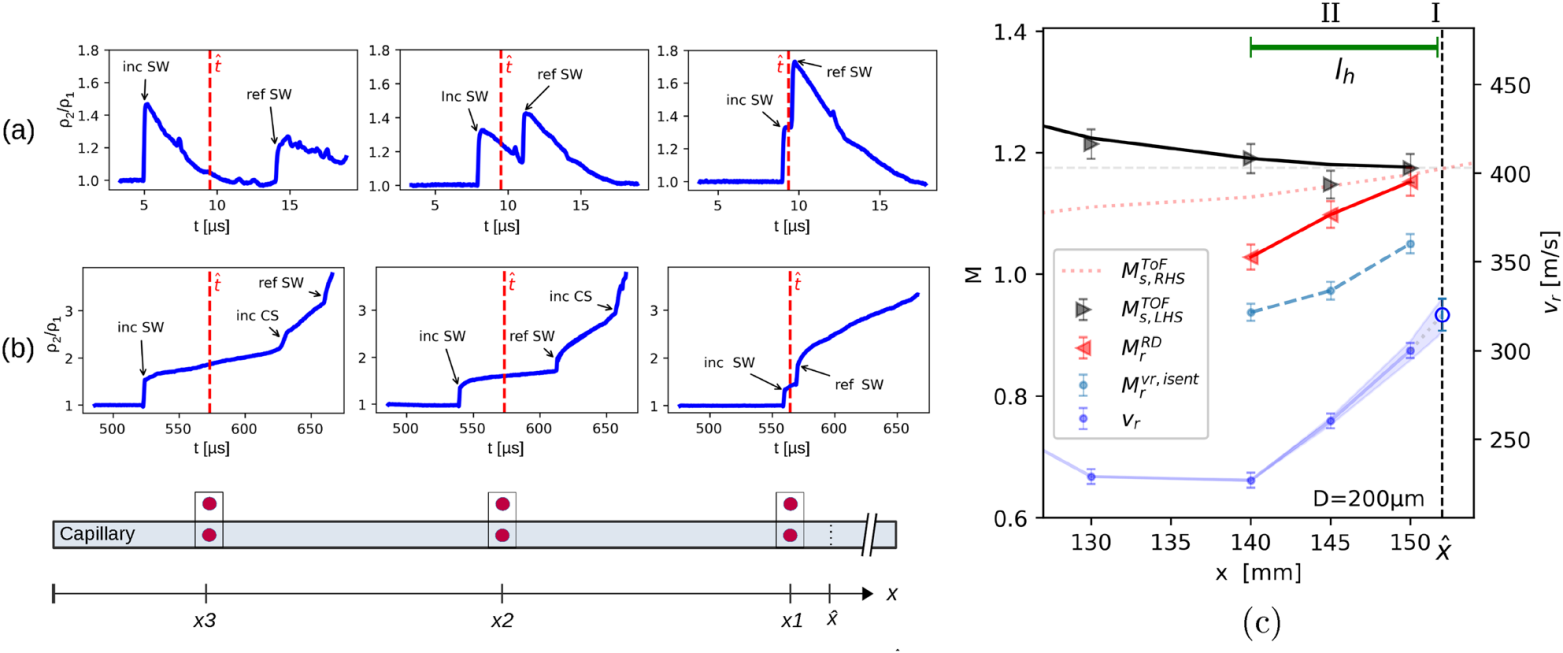
Results from colliding shock experiments with $\hat{M}_s \approx 1.20$: (**a**) unsteadily driven LIMS, (**b**) steadily driven VGMS. Evolution of the time-resolved density profiles at three different *x*-positions (RD-measurement). The time for the reflected shock (SW) to reach the related *x*-position increases from right to left. The contact surface (CS) is not always visible. (**c**) Results from steady shock reflection inside a $200\,\upmu \textrm{m}$ capillary with $\hat{M}_s = 1.19$ and a measured $\hat{M}_r = 1.18$. All lines in (**c**) are to guide the eye.

Later in time, for $t>\hat{t}$, the reflected shock wave travels from $\hat{x}$ to the LHS, in the direction of decreasing *x*-values. Initially, it enters the post-shock region of the incident shock, then propagates into the region affected by the rarefaction wave and eventually into a region where *p*, $\rho$ and *T* gradually return to their undisturbed values (see Fig. 4a, at position $x_3$). In order to investigate these regions, local thermodynamic state conditions have to be applied. This includes the transition from the non-isentropic adiabatic condition, described by RH at the shock front, to the isentropic expansion in the region significantly behind the shock^22^. Consequently, $u_{ps}$ and $T_{1r}=T_{2s}$ can be estimated by isentropic conditions for $x<\hat{x}$ (see Methods). Based on this, $\textrm{v}_r$ is converted to a Mach number, $M_{r}^{vr,isent}$, shown as dashed cyan line in Fig. 3. The convergence of both $M_{r}^{RD}$ and $M_{r}^{vr,isent}$ suggests a transition to an isotropic state.

Figure 3b shows data from the experiment repeated with a $D=100\,\upmu \textrm{m}$ capillary of $L=4.9\,\textrm{mm}$ with almost the same $\hat{M}_s^{ToF}=1.20$ and $\hat{M}_r^{RD}=1.19$. The ToF-measured velocity $\textrm{v}_r$ of the reflected shock extrapolates to $\hat{\textrm{v}}_r=317$ m/s. This is, when compared to the previous data, slightly lower since the marginally larger $\hat{M}_s$ implies a higher particle velocity towards $w_r$. The theoretical calculation by RH yields $\hat{u}_{ps}^{RH}=105$ m/s and $\hat{a}^{RH}_{2s}=364$ m/s from $\hat{T}^{RH}_{2s}=332$ K.

This, again, leads to consistent values of the theoretical $\hat{w}_{r}^{RH}=433$ m/s and the measured $\hat{w}_{r}^{vr,RH}~=~422$ m/s. In total, the reflection process in capillaries with $D=100\,\upmu \textrm{m}$ appears to be not much different to that in capillaries with $D=200\,\upmu \textrm{m}$. The complete parameter set, with error estimation, can be found in Table 1, for a detailed explanation of the nomenclature and related methods, the reader is referred to Table 3.

### Micro-shock collision and reflection: (b) differences between steady and unsteady driven shocks

Another important issue is the comparison of micro-shock waves in (quasi-)stationary and non-stationary processes. In general, a stationary process is defined as one in which the relevant quantities measured at a particular position *x* do not change with time *t*, or change only minimally in case of a quasi-stationary process. In contrast, for a non-stationary process, the quantities vary rapidly with *t* and are therefore functions of both *x* and *t*. For shock wave propagation, this distinction must be defined more carefully: Although shock waves imply rapid changes, specifically a pressure jump, this is not the criterion for the classification above. Instead, the classification depends on how *p*, $\rho$ and *T* behave behind the shock front and the decay of *M*(*x*, *t*). For the micro-shock waves considered in the present work, these different situations can be discriminated from Fig. 1c, d.

In case of LIMS, the fs-LPP is a very strong driver with an extremely short lifetime and hence an unsteady driver. Consequently, *p*, $\rho$ and *T* change rapidly behind the shock front (SW)^18^. This can be seen as a density tail which rapidly decays. The contact surface (CS) to the driver, if still strong enough, may be visible as a “second peak” (in Fig. 4a). The strong driver decay along with viscous losses leads also to a rapid decay of *M*(*x*, *t*), hence the propagation length for LIMS was limited to a few tens of millimeters.

In contrast, VGMS provides a constant reservoir pressure, producing a steadily driven shock wave throughout the propagation time until the shock wave reaches the end of the capillary. As a result, behind the shock front, there is a region of steady or gradually increasing density, which is typical for micro-shock waves with a steady driver. This density increase arises from the boundary layer and enhanced friction in general (see, e.g.^15,19^). The contact surface is always observed as a significant second “density jump”. The steady driver also leads to quasi-stationary evolution of *M*(*x*, *t*) (compare Fig 2a,b). This is the situation before the collision. The corresponding temporal density profiles of single-sided created shocks are displayed in Fig. 1c, d.

The profiles observed later in time, after the shock wave collision, are shown in Fig. 4 where the reflected shock wave enters into the regions described in the previous section. As the shock is reflected, it propagates back through the corresponding post-shock conditions. To illustrate this process, the temporal density profiles in Fig. 4a, b were taken for three different positions. Position $x_1$ is shortly after the reflection so that the reflected shock propagates into a region (❶, Fig. 1b) where the density is given by $\rho _{2s}(x,t)$ (similar for pressure and temperature). This is the post-shock density of the incident shock, i.e. region I. (note that experimental limitations prevent a measurement in the immediate vicinity of the front, i.e. for $|\hat{x}-x_1|\rightarrow 0$).

Position $x_2$ has been passed much earlier by the incident shock and later in time by the reflected shock front. Consequently, the reflected shock has now propagated further into the tail of the incident shock. Position $x_3$ was passed even earlier by the incident shock and even later by the reflected shock, so that the latter traveled even further in or even through the post-shock region of the incident shock. For better understanding, it is helpful to consider the time *t* at which the front of the incident and reflected shock arrive at the particular position $x_1$, $x_2$ or $x_3$, respectively.

The differences between the non-stationary and stationary conditions are obvious from Fig. 4. For a collision of unsteadily driven shocks, the reflected shock rapidly passes through the post-shock region before it enters the undisturbed region with laboratory environmental conditions. In contrast, for the collision of steadily driven shocks, the post-shock conditions of the incident shock largely persist until the reflected shock reaches the contact surface of the incident shock. Behind this (in region $\textcircled {3}$ in Fig. 1b), the test gas mixes with the driver gas, making the situation more complex. Moreover, due to the increased density resulting from the superposition of the incident and reflected shocks in region $\textcircled {3}$, the diagnostic limit is exceeded. This is not critical, as this region is not of main interest here; however, the short distance limits the available data points in-between.

Equivalent to Fig. 3, the data in Fig. 4c show the results of an experiment for the symmetric collision of VGMS inside a capillary with $D=200\,\upmu \textrm{m}$ and $L=300\,\textrm{mm}$. Although $M_r$ could only be measured until it enters the incident contact surface at $x<140$ mm, the three displayed data points are sufficient for the present analysis and discussion. Applying the same procedure of data analysis as before, the incident shock of Mach $\hat{M}_s^{ToF}=1.19$ yields $\hat{M}_r(\hat{M}_s)=1.18$ according to Eq. 5, which is consistent with the experimentally obtained $\hat{M}_r^{RD}=1.18$.

The ToF-measured velocity $\textrm{v}_r$ of the reflected shock (Fig. 3, blue) extrapolates to $\hat{\textrm{v}}_r=319$ m/s. RH predicts a particle velocity $\hat{u}_{ps}^{RH}=98$ m/s, which results in $w_{r}^{vr,RH}=417$ m/s. Further, with $\hat{a}_{2s}^{RH}$ and $\hat{T}^{RH}$ from RH, the reflected shock velocity becomes $\hat{w}_r^{RH}=426$ m/s. Considering the fluctuations in VGMS, the experimental data still agree with the theory. Again, the complete data set is listed in Table 1. Unlike unsteady-driven shocks, the velocity of the reflected shock ($\textrm{v}_r$) decreases after reflection, probably due to the positive density gradient between the shock front and the contact surface, caused by the boundary layer^6^.

### Reflected shock as a probe

It may be deduced from the previous sections that the reflected shock wave can be used as a probe of the local conditions of the incident shock and its post-shock region. This approach gives experimental access to the thermodynamic properties. That is of much importance, because, e.g., an experimental measurement of the temperature in shock wave experiments is rather difficult and still presents a challenge. Moreover, for the micro-regime up to now, the temperature at the shock front and behind has not been determined experimentally.

The reflected Mach number $M_r$ and the relative shock velocity $\textrm{v}_r$ are both linked to the local conditions, involving the temperature, as $M_r = w_r / a_{1r}$ with $a_{1r} = \sqrt{\gamma R T_{1r}}$. Immediately after the reflection or at the position $x=\hat{x}- \varepsilon$ (compare Fig. 1b), the conditions are $\hat{a}_{2s}=\hat{a}_{1r}$, $\hat{\rho }_{2s}=\hat{\rho }_{1r}$ and $\hat{T}_{2s}=\hat{T}_{1r}$. Because of the short time after the collision, heat diffusion and dissipation through the walls are assumed to be negligible. Therefore, the velocity $\hat{\textrm{v}}_r$ relates directly to the incident shock condition at $\hat{x}$, providing a supplementary measurement to check for consistency of experimental results and the applied theoretical model (see “Methods”).

In order to probe the temperature of the incident shock front as a function of *x*, and to obtain curves similar to *M*(*x*) (Fig. 2) or $\rho _{2}(x)$ from RD-measurements, $T_{2s}(x)$ can be determined through a series of measurements using different capillary lengths *L*. A variation of *L* leads to a change of $\hat{x}$ and $\hat{M}_s$ as well (see Fig. 2). Thus the reflected shock probes the incident shock always at $x= \hat{x}$ (details in Methods). As a demonstration, measurements were performed using capillaries with $D=100\,\upmu \textrm{m}$ and three different lengths and Mach number states. Again, it can be assumed that the experimental conditions allow to apply RH relations, which is reasonable since all Mach numbers remain relatively small.

The results are shown in Table 2 and imply a good consistence and therefore support the correctness of RH under the present conditions. The case of larger Mach numbers and/or other conditions is discussed further below. Moreover, from the ideal gas equation, the full set of $\rho _{2s}$, $T_{2s}$ and $p_{2s} = (\rho R T_{2s})/M_m$ ($M_m$: molar mass, *R*: universal gas constant) can be calculated for any given position without an additional measurement of the pressure. Thus one can also take advantage of the spatial and temporal resolution of the optical diagnostics, which is mostly superior to that of the typically applied pressure sensors.

**Table 1:** Results from LIMS and VGMS collision experiments by different methods in capillaries of diverse diameters and lengths.

*D*	VGMS	LIMS	LIMS	[$\upmu$m]
	$200\pm 5$	$200\pm 5$	$100\pm 3$	
*L*	$304 \pm 2.0$	$8.3\pm 0.1$	$4.9 \pm 0.1$	[mm]
$\hat{M}_s^{TOF}$	$1.19\pm 0.01$	$1.19\pm 0.01$	$1.20 \pm 0.01$	-
$\hat{M}_r(\hat{M}_s)$	$1.18\pm 0.01$	$1.18\pm 0.01$	$1.19 \pm 0.01$	-
$\hat{M}_r^{RH}$	$1.18\pm 0.01$	$1.18\pm 0.01$	$1.19 \pm 0.01$	-
$\hat{\textrm{v}}_r$	$319\pm 10$	$320\pm 10$	$317 \pm 10$	[m/s]
$\hat{u}_{ps}^{RH}$	$98\pm 3$	$101\pm 3$	$105 \pm 3$	[m/s]
$\hat{w}_r^{\textrm{v}_r,RH}$	$417\pm 11$	$421\pm 11$	$422 \pm 11$	[m/s]
$\hat{w}_r^{RH}$	$426\pm 6$	$429\pm 6$	$433 \pm 7$	[m/s]
$\hat{T}_{2s}^{RH}$	$329\pm 4$	$330\pm 4$	$332 \pm 5$	[K]

**Table 2:** Results from the analysis of shock probe experiments (LIMS) for three different shock collision Mach numbers and positions inside a $100$ $\upmu$m capillary. The laser intensity was fixed to $I_L=3\times 10^{12}\mathrm {W/cm^2}$.

*L*		${M=1.20}$	${M=1.38}$	${M=1.44}$	[mm]
		${\hat{x}_1=2.46 {\rm mm}}$	${\hat{x}_2=1.47 {\rm mm}}$	${\hat{x}_3=1.05 {\rm mm}}$	
		$4.9 \pm 0.1$	$3 \pm 0.1$	$2 \pm 0.1$	
$\hat{M}_s^{TOF}$		$1.20 \pm 0.01$	$1.38\pm 0.01$	$1.44\pm 0.01$	-
$\hat{M}_r(\hat{M}_s)$	Eq. 5	$1.19\pm 0.01$	$1.36\pm 0.01$	$1.39\pm 0.02$	-
$\rho _{2s}/\rho _{1s}$	RD, Fig. 2	$1.34\pm 0.02$	$1.65\pm 0.02$	$1.76\pm 0.03$	-
$p_{2s}/p_{1s}$	Ideal gas eq.	$1.51\pm 0.04$	$2.05\pm 0.05$	$2.25\pm 0.06$	-
$T_{2s}/T_{1s}$ | $\hat{T}_{2s}^{RH}$	RH only	$1.11 | 332\pm 4$	$1.24 | 368\pm 5$	$1.28 | 380\pm 5$	- | [K]
$T_{2s}/T_{1s}$ | $\hat{T}_{2s}^{a,vr}$	Shock probe	$1.08 | 323\pm 7$	$1.26 | 375\pm 9$	$1.28 | 380\pm 9$	- | [K]
$\hat{w}_r = \hat{\textrm{v}}_r - \hat{u}_{ps}^{RH}$	Eq. 7	$422\pm 11$	$524\pm 15$	$542\pm 16$	[m/s]
$\hat{a}_{2s}^{RH}$	RH only	$364 \pm 5$	$384\pm 6$	$389\pm 6$	[m/s]
$\hat{a}_{2s}^{\hat{\textrm{v}}r,RH}$	Shock probe	$361\pm 11$	$388\pm 12$	$390\pm 12$	[m/s]

**Table 3:** Comprehensive overview of the nomenclature used for quantities to annotate origin (subscript) and the used model or approach (superscript).

Label	Origin / model
$M_s^{ToF}$, $M_{s,LHS}^{ToF}$	From ToF-measurement with $a_{1s}$
$M_s^{RD}$	From RD-measurement with RH (Eq. 2)
$M_r(M_s)$	from $M_s^{ToF}$ with RH related Eq. 5
$M_r^{RD}$	From RD-measurement with RH (Eq. 2)
$M_r^{\textrm{v}r,isent}$	From $\textrm{v}_r$ + $u_p^{isent}$ (Eq. 11) and $a_{1r}^{isent}$
$M_r^{vr,RD}$	From $\textrm{v}_r$ + $u_p^{RH}$ (Eq. 7) and $a_{1r}^{RH}$
$u_{ps}^{RH}$	Particle velocity from RH conditions (Eq. 2)
$u_{ps}^{isent}$	Particle velocity from isentropic conditions (Eq. 11)
$w_s^{ToF}$	shock velocity directly obtained from the ToF measurement with u$_{ps}$=0
$\textrm{v}_r$	Relative shock velocity from ToF-measurement with $u_{ps} \ne 0$
$w_r^{vr,isent}$	Reflected shock velocity from $\textrm{v}_r + u_p^{isent}$
$w_r^{vr,RH}$	Reflected shock velocity from $\textrm{v}_r + u_p^{RH}$
$w_r^{RH}$	Reflected shock velocity from $M_r^{RD}$ and $a_1r$
$T_{2s}^{a,vr}$	Temperature based sonic speed $a=a_r=a_s$, by $w_r^{vr,isent}$ and $M_r(M_s)$ at $\hat{x}$ (Eq. 13)
$T_{2s}^{RH}$	Temperature based on Mach Number (Eqs. 2,12)
$a_{2s}^{vr,RH}$	Sonic speed obtained by $w_r^{vr,isent}$ and $M_r(M_s)$ at the probing point $\hat{x}$ (Eq. 4)
$a_{2s}^{RH}$	Sonic speed based on RH calculated temperature (Eqs. 4,12)

## Discussion

The collision of shock waves in small capillaries with inner diameter of $D=200\,\upmu \textrm{m}$ and $D=100\,\upmu \textrm{m}$ was investigated experimentally. In particular, the density of the incident shock wave was measured at different spatial positions *x*. Because at each *x*-value its temporal evolution was recorded, in total the whole profile, i.e., the shock front and the complete post-shock region, could be observed. Additionally, the complete density (and potentially temperature) profile was obtained from the analysis of the reflected shock data, as discussed in Results, “micro-shock collision and reflection” and below. Such a profile at $x_s$ ($=\hat{x}$) could also be obtained for any other value by choosing $\hat{x}$ accordingly (“Results”, “Reflected shock as a probe”). Consequently, the profile could be determined at any position and for any time, at least in principle.

Figures 3 and  4b show the most important results: The Mach number of the reflected shock wave was deduced from the measured density data and the direct velocity measurements of the reflected and the incident shock wave Mach number. The analysis provides a reliable cross-check that RH relations are applicable for the low Mach number of the incident shock wave at the collision position. The low value of $M_s$ was deliberately chosen for this reason (high temperatures and real gas effects were avoided). Within the experimental error, all deduced values of $M_r$ are in good agreement (for LIMS measurements, see Fig. 3; for the VGMS experiment see Fig. 4c). For the VGMS data, the calculated scaling parameter $S_c$ is close to unity, further supporting these results. This may be regarded as proof that the measurement methodology, its implementation, and the data analysis work well.

In addition, the theoretical temperature profile was verified by analyzing the reflected shock data. At the shock front (region I), the reflected shock experiences the local conditions, such as $T_{2s}$, of the incident shock front, where RH is assumed to be valid. The reflected shock velocity $w_r$ is obtained by correcting $\textrm{v}_r$ with $u_{ps}^{RH}$ from RH relations (Eq. 7). Using $w_r$ to obtain the sonic speed from $\hat{M}_r^{RH}$, the incident shock temperature was then calculated. The direct approach using only RH relations (Eqs. 2) derived the same temperature within the measurement error.

To determine the temperature at any position along the shock propagation, in principle, $\hat{x}$ can be shifted by changing the capillary length *L*. Then, since the pressure *p* can be obtained from $\rho$ and *T*, a characterization of the shock region of the incident shock wave or, more generally, for a micro-shock wave propagating through a small capillary, becomes feasible. This is exemplified in Table 2.

For potentially larger Mach numbers of the incident shock the correct description of the micro-shock using RH relations cannot necessarily be expected. In such a case, the assumption of a loss-less process may no longer hold. In particular, due to wall-friction, viscous and heat effects that are significant, especially for micro-shock waves. Smaller capillary diameters and/or lower pressures, will strongly affect boundary layer effects. Potentially, the (local) Knudsen number may become larger, indicating a transition to the ballistic regime. Consequently, this would also change the flow characteristics. Then the ideal RH equations may not be applicable, but they must be modified accordingly. At present, no general theory for micro-shock waves is available.

However, numerical simulations of shock tubes operated at very low pressure ($p_1=10\,\textrm{Pa}$) suggest significant deviations in the local temperature from RH theory ^7^. Results from future theoretical and numerical work on that topic may be confirmed with data from the current and future collision experiments. In particular, the particle flow velocity $u_{ps}$ has to be described properly to match the experimentally measured value of $\textrm{v}_r$ and the deduced value $w_r$. The related temperature evolution $T_{2s}(x)$, along with the directly measured $\rho _{2s}(x)$ and $M_s(x)$ evolution, would need to be well described by a theoretical model or a simulation. In this sense, the reflected shock may act as a probe and a proof of current and newly developed models.

The present work already leads to open questions: Although there is a good agreement for $M_r$ from the RD-measurement data and those from the ToF-measurement at $\hat{x}$ (region I; see Fig. 2), and also in the region III (for $D=100\,\upmu \textrm{m})$, this is not the case for region II (compare the data displayed in cyan and red). For even smaller *x*-values (region IV), the reflected shock enters the region of the incident rarefaction wave. Here again, all experimental data agree. In that region the particle flow is in opposite direction so that the necessary correction for the velocity must be $w_r = \textrm{v}_r - u_{ps}$ (with absolute values). Without that correction, the “overshooting” of $\textrm{v}_r$ may be observed.

For LIMS and the currently low Mach numbers of the incident shocks, this means that the reflected shocks are well described at the collision position. There the shock leads to discontinuity of *p*, $\rho$ and *T* and an increase of entropy. The local thermodynamic condition is adiabatic and non-isentropic, and thus RH relations are applicable. The reflected shock is well described also much before in space (i.e., much later in time), when the reflected shock has reached the rear part of the post-shock region of the incident one (region III). Here the thermodynamic states change more smoothly; There is a quasi-equilibrium and the flow is continuous and can be described by isentropic relations. Both conditions, adiabatic and isentropic, set different relations for *p*, $\rho$, *T*, $u_{p}$, etc.

The physical reason for the deviation from isentropic conditions in region II is a complex combination of dissipative effects, including heat transfer and viscous (momentum-related) dissipation. One may argue that this should lead to an overcompensation of $\textrm{v}_r$ by $u_{ps}$ from an isentropic model ^6^, however for low Mach numbers $u_{p}^{isent}$ is still smaller than $u_{p}^{RH}$ (see Methods). In particular, the boundary layer may play a considerable role. The ratio of boundary layer thickness to tube diameter *D* increases significantly as *D* decreases ^7,9,12,14,18,20,34^. A boundary layer will also build up in LIMS, however the very short post shock decay over 5$\upmu$s or less will result in a much smaller extend than in VGMS.

Potentially, a hint from the present work regarding boundary layer effects for LIMS may be observed from Fig. 3. Although in case of the $D=100\,\upmu \textrm{m}$ capillary, the extension of region II is smaller in absolute values when compared to the $D=200\,\upmu \textrm{m}$ capillary, it also seems slightly shorter when normalized to *D*. That, in turn, may indicate the relative increase of the boundary layer thickness in relation to the capillary diameter as the dissipation effects influence the transition to isentropic conditions. A more quantitative analysis and potentially measurements with other capillary diameters remains as task and could be regarded as a motivation for future theoretical work. Furthermore, a general analytical theory applicable to micro-shock waves would be highly valuable.

For the steadily driven shock (VGMS), the related discussion is obviously restricted to the hot flow region $l_h$ which is displayed in Fig. 4c. The analysis is the same as before, but for VGMS the density behind the shock increases (partially this might be enhanced in the measured signal by some boundary build-up), whereas it decreases for LIMS. Consequently, $u_{ps}$ rapidly slows down behind the shock front in the case of a non-stationary process, whereas it increases as it gets closer to the contact surface in case of a quasi-stationary condition. Thus, performing the correction of $\textrm{v}_r$ properly, one obtains $w_r$, shown as the cyan data in Fig. 4c. Irrespective of this, there is again a mismatch with the data displayed in red.

It can be concluded that the present work demonstrates the need for a more detailed investigation of the hot flow region II, particularly through theoretical analysis. Indeed, the importance of the hot flow region has been recognized once again in a recent work on computational fluid dynamics, which focused on “Post-shock flow in micro-channels” ^28^.

We highlight this, as the hot flow region is not only of fundamental interest but also of particular importance for many applications, where it serves as a test region. This is well known in experiments conducted with large-scale shock tubes. In the micro-range this is undoubtedly important as well. Due to the large common interest in microfluidics and micro-electro-mechanical systems (MEMS) in general, microfluidic technologies, as well as biological and medical devices, are growing in importance. This creates a clear need for a deep understanding of the underlying physics for these interdisciplinary applications. A crucial part of this understanding involves the well-characterized hot flow region, where, for example, chemical reactions may occur in a micro-shock tube.

Besides its relevance to applications, the present work contributes significantly to the field of shock and blast waves in general. In this sense, it should be noted that experiments with micro-shock waves provide a much simpler and more cost-effective approach compared to traditional experiments conducted with large-scale shock tubes. While micro-shock waves will not replace macro shock waves, this work highlights them as a valuable alternative. For instance, with a large-scale tube, it is difficult to achieve a head-on collision of counter-propagating shocks with exactly the same strength at a precise position in the tube. Thus, the present work establishes well-controlled and clearly defined conditions with high flexibility, making it a viable option when these issues are relevant. A variety of experimental conditions could be easily arranged, including variations in pressure, tube diameter, length, etc., as well as the type of driver and driver strength on both sides of the “tube”. Thus, experiments with asymmetric shocks, or those where one side is driven by a steady driver and the other by an unsteady one, require minimal effort. This presents significant potential for future investigations.

In conclusion, the present work reports on the first experiments with counter-propagating micro-shock waves and their head-on shock collision in a micro-capillary. The collision was symmetric and therefore established ideal reflection conditions. Moreover, these experiments are the first to compare the collision of steadily and unsteadily driven (micro-) shock waves. Thus, the present work contributes not only significantly to the field of shock and blast waves in general, but also to the hot topic of “micro-shock wave physics and technology”, in particular.

## Methods

### Diagnostics

The main diagnostics used in the present work is a laser interferometer. In case of the RD-measurement, when a shock wave passes through the region traversed by the probe beam (Fig. 1a), the density changes. Due to the density dependency of the refractive index, this causes a change in the optical path length. In turn, this change causes a shift $\Delta \phi$ of the optical phase relative to the reference beam, which passes through the region above the capillary where $\rho _{\text {ref}} = \text {const}$ (here $\rho _{\text {ref}} = \rho _1$). By applying the Gladstone-Dale relation, the density $\rho$ in the probed region could be obtained. A measurement of $\Delta \phi (t)$ yields $\rho (t)/\rho _1$. The maximum measurable density is limited by $\Delta \phi = \pi$, which, in the present experiments, was only exceeded in regions behind the contact surface. However, this region $\textcircled {3}$ was not of interest here. As the interferometer works at the center height but still passes through two side walls with potential boundary layers, those may add to the signal. However, the data analysis relies on the (relative) jump at the shock front. In addition, the boundary layer requires time to develop, thus it is only noticeable in the VGMS post shock towards the contact surface.

In case of the ToF-measurement, both interferometer beams traverse the capillary. When the shock wave passes either of the beams, it initiates a phase change with respect to the other one, which can be easily detected. The same occurs when the second beam is passed. From the time difference and the distance $\Delta x_{\text {ToF}} = (354 \pm 2)\,\upmu \text {m}$, $w_s$ and $\textrm{v}_r$ are obtained directly. For details of the instrument and its operation see, e.g.,^ 18,19,33^.

### Mach number of the reflected shock

Most relevant for shock wave related calculation are the three fundamental equations of conservation (Eq. 1) along with the RH relations for *p*, $\rho$ and *T* (Eq. 2):

1 $$\begin{aligned} & \begin{aligned} \text {Mass:}&\quad \rho _1 u_{p1} = \rho _2 u_{p2} \\ \text {Momentum:}&\quad p_1 + \rho _1 u_{p1}^2 = p_2 + \rho _2 u_{p2}^2 \\ \text {Energy:}&\quad h_1 + \frac{1}{2} u_{p1}^2 = h_2 + \frac{1}{2} u_{p2}^2 \end{aligned} \end{aligned}$$

2 $$\begin{aligned} & \begin{aligned} \frac{p_2}{p_1}&= 1 + \frac{2\gamma }{\gamma +1}\left( M^2 - 1\right) \\ \frac{\rho _2}{\rho _1}&= \frac{(\gamma +1)M^2}{2 + (\gamma -1)M^2} \\ \frac{T_2}{T_1}&= \frac{\nicefrac {p_2}{p_1}}{\nicefrac {\rho _2}{\rho _1}}= \left( 1 + \frac{2\gamma }{\gamma +1}(M^2 - 1)\right) \cdot \frac{2 + (\gamma -1)M^2}{(\gamma +1)M^2} \end{aligned} \end{aligned}$$

The calculation of the reflected Mach number $M_r$ from the incident $M_s$ assumes an ideal reflection, meaning no energy dissipation. Under the present condition of two identical shocks at low Mach numbers, this is a good approximation. The second assumption arises from the symmetry of the process and dictates, that the particle velocity $\hat{u}_p$ at the collision point $\hat{x}$ must be zero.

Therefore, both particle velocities, $u_{p1}$ and $u_{p2}$, from the *LHS* and *RHS* shock must be equal. Consequently, mass and momentum conservation from Eqs. 1 are applied along with the RH relations from Eqs. 2 to obtain an expression for $u_{p1}$ and $u_{p2}$ , respectively, as shown by Eq. 6 below. Conclusively, rearranging the equation for mass conservation leads to the following expression:

3 $$\begin{aligned} \frac{M_s^2-1}{M_s} a_{1s} = \frac{M_r^2 -1}{M_r} a_{2s} \implies \frac{M_r}{M_r^2 -1} = \frac{M_s}{M_s^2 -1}\cdot \frac{a_{1s}}{a_{2s}} \end{aligned}$$

where $a_{1s}$ is the sonic speed before and $a_{2s}$ the sonic behind the shock front of the incident shock. Using the general relationship for the sound velocity:

4 $$\begin{aligned} a=\sqrt{\gamma R_s T} \end{aligned}$$

where $\gamma$ is the adiabatic constant (1.4 for air) and $R_s$ the specific gas constant, the relation $\nicefrac {a_{1s}}{a_{2s}}$ is replaced by $\sqrt{\nicefrac {T_1}{T_2}}$ from Eq. 2, yielding the following equation as given in^22^:

5 $$\begin{aligned} \boxed {\frac{M_r}{M_r^2 - 1} =\frac{M_s}{M_s^2 - 1} \, \sqrt{1+\frac{2(\gamma -1)}{(\gamma +1)^2} ( M_s^2-1)\left( \gamma +\frac{1}{M^2_s}\right) }} \end{aligned}$$

### Particle velocity

The ToF-measurements provides a shock wave velocity relative to the resting laboratory frame. However, the reflected shock propagates into a region where the particle velocity $u_p \ne 0$. Therefore, these velocities need to be corrected. Depending on the local conditions either RH or isentropic relations were applied.

*Adiabatic, non-isentropic (RH) particle velocity*: as the conservation of mass applies (see Eq. 1), the mass flow before and behind the shock must be the same. Since a dependence on *M* is preferred in the shock wave context, we will use the RH relation of $\nicefrac {\rho _2}{\rho _1}$ from Eq. 2 directly instead of the momentum equation. To relate the flow to the shock Mach number, $u_{p1}$ is now the shock speed, expressed by $M_s$ and the sonic speed $a_{1s}$ in front of the shock: $u_{p1}=w_s=a_{1s} M_s$. The equation for mass conservation then yields:

6 $$\begin{aligned} \rho _1 \cdot a_{1s}\cdot M_s = \rho _2 \cdot u_{p2} \implies u_{p2} = a_{1s} \cdot M_s \cdot \frac{\rho _1}{\rho _2} \end{aligned}$$

and with the RH relation for $\nicefrac {\rho _2}{\rho _1}$, the particle velocity right behind the shock front becomes:

7 $$\begin{aligned} u_{p2} = a_{s1} \cdot M_s \frac{(\gamma -1)M^2_s+2}{(\gamma +1) M^2_s} \implies \boxed {u_{p}^{RH} = \frac{2 a_{s1}}{\gamma +1}\frac{M^2_s -1}{M_s}} \end{aligned}$$

*Isentropic particle velocity*: the particle velocity for an isentropic expansion is driven by the pressure gradient. However, from the interferometric measurement only $\rho$ is accessible. For isentropic conditions $p\propto \rho ^\gamma$ and $T \propto \rho ^{\gamma - 1}$. Using the ideal gas law, for $\nicefrac {p_2}{p_1}$, one gets:

8 $$\begin{aligned} p_1 = \rho _1 R_s T_1 \quad \text {and} \quad p_2 = \rho _2 R_s T_2 \end{aligned}$$

Substituting with the isentropic relation for $T_2$ and $T_1$ from above, results in:

9 $$\begin{aligned} \frac{p_2}{p_1} = \frac{\rho _2}{\rho _1} \cdot \left( \frac{\rho _2}{\rho _1} \right) ^{\gamma - 1} = \left( \frac{\rho _2}{\rho _1} \right) ^\gamma \end{aligned}$$

Using the energy conservation from Eq. 1 with the specific enthalpy $h=C_p T$, where $C_p$ is the heat capacity at constant pressure, this yields:

10 $$\begin{aligned} C_P T_1 + \frac{u_{p1}^2}{2} = C_P T_2 + \frac{u_{p2}^2}{2} \end{aligned}$$

Using the temperature-density relation from before, the equation can be rearranged for the isentropic particle velocity:

11 $$\begin{aligned} \boxed {u_{p}^{isent} = \frac{2 a_2}{\gamma - 1} \left[ \left( \frac{\rho _2}{\rho _1} \right) ^{\tfrac{\gamma - 1}{2}} - 1 \right] } \end{aligned}$$

where $a_{s2}$ depends on the isentropic calculated temperature (in the region behind the shock front).

### Shock probing

Supplementary to the direct measurement of $\nicefrac {\rho _{2r}}{\rho _{1r}}$, the measurement of $\textrm{v}_r$ in the laboratory frame provides additional information on the temperature and other state conditions close to the front of the incident shock and further behind. The thermodynamic state quantities and their relations to each other have to be described by an appropriate theoretical model. In the present case of low Mach numbers, the condition at the shock front is well described by the adiabatic condition and the RH relations.

Thus, the theoretical temperature can be obtained directly from the experimental collision Mach Number $\hat{M}_s^{RD}$ using (Eq. 2):

12 $$\begin{aligned} \boxed {T_{2s} = T_{1s} \, \frac{ \left( 1 + \frac{2\gamma }{\gamma +1}(M_s^2 - 1)\right) }{ \rho _{2r}/\rho _{1r} } } \end{aligned}$$

In the direct vicinity of the collision (region $\hat{x}- \varepsilon$), the following conditions are considered to be valid $\hat{a}_{2s}=\hat{a}_{1r}$, $\hat{\rho }_{2s}=\hat{\rho }_{1r}$ and $\hat{T}_{2s}=\hat{T}_{1r}$ (compare Fig. 1). This assumption, along with an adequate model, allows to correct the measured velocity $\textrm{v}_r$ with the incident shocks particle velocity. In case of the present work, this is $u_{ps}^{RH}$ from Eq. 7, which leads to $w_r$. With the calculated reflected Mach number $\hat{M}_r(\hat{M}_s)$ from Eq. 5 and the general relation $M=w/a$, the local sonic speed $a_{1r}=a_{2s}$ is obtained. Rearranging Eq. 4 for $T_{2s}$ then yields:

13 $$\begin{aligned} \boxed {T_{2s}^{a,vr} = \frac{a_{2s}^2}{\gamma R} \quad \text {or}\quad T_{2s,vr} = T_{1s} \left( \frac{a_{2s}}{a_{1s}} \right) ^2} \end{aligned}$$

## Data Availability

The datasets generated and analyzed during the current study are available from the corresponding author on reasonable request.
